# Analysis of blood untargeted metabolomic characteristics of different subtypes of rosacea

**DOI:** 10.3389/fmolb.2025.1652995

**Published:** 2025-11-27

**Authors:** Kejia Zhang, Yanyan Feng, Xiaoyu Zhang, Xingyan He, Sichun Qin, Xinxian Hu, Yuxi Liang

**Affiliations:** 1 Department of Dermatology, Chengdu Second People’s Hospital, Chengdu, Sichuan, China; 2 Department of Epidemiology, Nanjing Medical University, Nanjing, Jiangsu, China

**Keywords:** erythematotelangiectatic rosacea, papulopustular rosacea, untargeted metabolomics, skin physiological parameters, metabolomics

## Abstract

**Background:**

Rosacea is a chronic inflammatory skin disease characterized by vascular and neurological dysregulation, presenting with diverse clinical subtypes whose pathological mechanisms remain incompletely elucidated. Recent studies suggest that metabolic dysregulation may play a key role in disease onset and progression; however, systematic metabolomic studies targeting different subtypes remain limited.

**Objective:**

This study employed untargeted metabolomic analysis to systematically compare plasma metabolic characteristic differences between patients with erythematotelangiectatic rosacea (ETR), papulopustular rosacea (PPR), and healthy controls (HC), aiming to identify potential disease biomarkers and provide new insights for understanding the pathogenesis of rosacea.

**Methods:**

Ultra-high performance liquid chromatography-mass spectrometry (UPLC-MS) was used to compare metabolic profiles of plasma samples from ETR, PPR, and HC groups. Key differential metabolites identified were subjected to correlation analysis with disease severity and skin physiological parameters.

**Results:**

ETR patients primarily involved amino acid metabolism, carbon metabolism, and cholesterol metabolism pathways, with key metabolites including upregulated SSA and 2,3-DHPA, and downregulated TCDCA and Met. PPR patients primarily involved tryptophan and linoleic acid metabolism pathways, with key metabolites including upregulated 12-HSA, DGLA, and 5-ALA, and downregulated 5-HTP and 3-HPPA. Metabolic differences between different rosacea subtypes were associated with steroid hormone biosynthesis. DGLA showed positive correlation with disease severity, while 5-HTP showed negative correlation with disease severity. Met was closely related to skin barrier function. Both 12-HSA and DGLA showed positive correlation with sebum secretion.

**Conclusion:**

These findings elucidate the metabolic characteristics of rosacea and their associations with disease severity and skin physiological parameters, providing new theoretical foundation and potential targets for subtype diagnosis and precision treatment of the disease.

## 1 Introduction

Rosacea is a chronic inflammatory skin disease primarily affecting facial blood vessels and pilosebaceous units in the central facial region, which can be classified into four subtypes: erythematotelangiectatic rosacea (ETR), papulopustular rosacea (PPR), phymatous rosacea (PHR), and ocular rosacea (OR) (Crawford et al., 2004). A global study in 2024 showed that the overall prevalence of rosacea reached 5.1%, with the 25–39 age group showing higher disease risk compared to other age groups (Saurat et al., 2024). The pathogenesis of rosacea remains incompletely understood, with current research suggesting that it essentially represents a multifactorial-mediated imbalance in the “neuro-vascular-immune-barrier” network (Geng et al., 2024).

Metabolomics can provide a comprehensive metabolic landscape of the body under different physiological and pathological states, contributing to a deeper understanding of disease molecular mechanisms (Anh et al., 2024). Based on research objectives, metabolomics can be divided into untargeted metabolomics and targeted metabolomics (Cambiaghi et al., 2017). Compared to targeted metabolomics, which focuses on validating specific pathways, untargeted metabolomics is more suitable for discovering novel disease biomarkers and unknown metabolic regulatory mechanisms (Qiu et al., 2023). In recent years, metabolomics has achieved new breakthroughs in the diagnosis and treatment research of skin diseases such as psoriasis, atopic dermatitis, and melanoma (Paganelli et al., 2022). Metabolomics technology provides new perspectives and tools in dermatological disease research, helping to better understand the complex pathophysiological processes of diseases.

An increasing number of studies suggest that metabolic dysregulation is an important risk factor for rosacea development, but the exact causal relationship between metabolic abnormalities and disease onset and progression remains incompletely elucidated. Existing metabolomics studies have been limited to local skin (Ní et al., 2012) or blood targeted detection (Liu et al., 2022), lacking systematic blood untargeted metabolic profiling analysis, particularly with knowledge of metabolic heterogeneity among different clinical subtypes remaining a blank field.

## 2 Materials and methods

### 2.1 Sample collection

From November 2023 to March 2024, Thirty-four rosacea patients (17 ETR cases, 17 PPR cases) and 16 age-, sex-, and BMI-matched healthy controls were recruited from the Department of Dermatology at Chengdu Second People’s Hospital. Rosacea patients were required to meet the diagnostic criteria for rosacea established by the Global Rosacea Consensus Panel in 2019 (Schaller et al., 2020), with diagnosis confirmed by two dermatologists. Exclusion criteria for all participants included: systemic diseases and other skin conditions, history of systemic immunomodulators or antibiotics, intake of prebiotics or probiotics, extreme diet within the past 12 weeks, pregnancy and lactation.

Disease severity of enrolled rosacea patients was assessed using the Clinician Erythema Assessment (CEA) scale (Logger et al., 2020). Venous blood (2 mL) was collected from all subjects after fasting for 12 h in routine biochemical coagulation tubes, centrifuged, and stored at −80 °C until analysis.

Non-invasive skin physiological parameter measurements were performed on all subjects to assess stratum corneum hydration, transepidermal water loss (TEWL), and sebum content on bilateral cheeks (measurements taken 30 min after cleansing while seated, at ambient temperature 18 °C–22 °C and relative humidity 40%–60%; final data represented the average of three consecutive measurements for each parameter).

This study strictly adhered to the ethical principles of the Declaration of Helsinki, and the protocol was approved by the Ethics Committee of Chengdu Second People’s Hospital (Ethics No.: 2023402). All participants provided written informed consent.

### 2.2 Sample preparation

Serum samples stored at −20 °C (including sample preparation QC) were thawed in a 4 °C refrigerator until no ice crystals were visible. One hundred microliters of each sample was transferred to EP tubes, with remaining samples refrozen. Seven hundred microliters of extraction solvent (methanol:acetonitrile:water = 4:2:1, v/v/v) was added, vortexed for 1 min, and incubated at −20 °C for 2 h. Samples were centrifuged at 25,000 g at 4 °C for 15 min. Six hundred microliters of supernatant was transferred to new EP tubes and dried using a vacuum concentrator. Samples were reconstituted with 180 μL methanol:water (1:1, v/v), vortexed for 10 min until completely dissolved, and centrifuged at 2,500 g at 4 °C for 15 min. Supernatant was transferred to new EP tubes. Quality control (QC) samples were prepared by pooling 20 μL from each sample, and prepared supernatants were analyzed.

### 2.3 Untargeted metabolomics analysis

#### 2.3.1 Analytical platform

Untargeted metabolomics analysis was performed using a Waters UPLC I-Class Plus (Waters, United States) coupled with a Q Exactive high-resolution mass spectrometer (Thermo Fisher Scientific, United States).

#### 2.3.2 Chromatographic conditions

BEH C18 column (1.7 μm, 2.1 × 100 mm, Waters, United States) was used for chromatographic separation. For positive ionization mode, the mobile phases were 0.1% formic acid in water (mobile phase A) and 0.1% formic acid in methanol (mobile phase B). For negative ionization mode, the mobile phases were 10 mM ammonium formate in water (mobile phase A) and 10 mM ammonium formate in 95% methanol (mobile phase B). The gradient elution was as follows: 0–1 min, 2% B; 1–9 min, 2%–98% B; 9–12 min, 98% B; 12–12.1 min, 98%–2% B; 12.1–15 min, 2% B. The flow rate was 0.35 mL/min, column temperature was 45 °C, and injection volume was 5 μL.

#### 2.3.3 Mass spectrometric acquisition

Q Exactive mass spectrometer (Thermo Fisher Scientific, United States) was used for MS1 and MS2 data acquisition. The mass spectrometric scan range was m/z 70–1,050, with MS1 resolution of 70,000, AGC target of 3e6, and maximum injection time (IT) of 100 ms. Based on precursor ion intensity, the top 3 ions were selected for fragmentation to acquire MS2 information, with MS2 resolution of 17,500, AGC target of 1e5, maximum injection time (IT) of 50 ms, and stepped normalized collision energy (NCE) set at 20, 40, and 60 eV. Electrospray ionization (ESI) source parameters were set as follows: sheath gas flow rate of 40, auxiliary gas flow rate of 10, spray voltage of 3.80 kV (positive mode) and 3.20 kV (negative mode), capillary temperature of 320 °C, and auxiliary gas heater temperature of 350 °C.

### 2.4 Data processing and statistical analysis

#### 2.4.1 Statistical analysis of clinical data

Statistical analysis was performed using SPSS 25.0 software, with *P* < 0.05 considered as the criterion for statistical significance. Data following normal distribution were expressed as mean ± standard deviation, while data with skewed distribution were expressed as median (interquartile range). For comparisons of quantitative data among three groups, one-way analysis of variance (ANOVA) was used for normally distributed data, while the Kruskal-Wallis H test was applied for skewed distribution data. For comparisons of quantitative data between two groups, independent samples t-test was used for normally distributed data, while the Wilcoxon rank-sum test was used for data with skewed distribution.

#### 2.4.2 Metabolomic data analysis

Raw data processing was performed using Compound Discoverer 3.1 software (Thermo Fisher Scientific), including peak extraction, retention time correction, adduct identification, peak area integration, and background noise removal. Data preprocessing applied an 80% rule filter (i.e., peaks detected in at least 80% of samples in at least one group were retained), missing values were imputed using the K-Nearest Neighbors (KNN) method (which estimates missing values based on the similarity patterns of the k most similar samples), and systematic errors were corrected using probabilistic quotient normalization (PQN) method after log transformation.

QC samples were prepared by mixing equal volumes of all study samples, with one QC sample inserted every 10 study samples. Isotopically labeled internal standards (d3-serine, 13C6-leucine, etc.) were added for quantitative correction. A relative standard deviation (RSD) <30% for metabolite peak areas in QC samples was used as the data quality criterion.

Metabolite identification was performed strictly following the Metabolomics Standards Initiative (MSI) guidelines for confidence level classification. Metabolite structural identification was conducted using the Human Metabolome Database (HMDB), Kyoto Encyclopedia of Genes and Genomes (KEGG), BGI high-resolution metabolomics reference standards database (BMDM 3.0), and mzCloud online reference standards database.

Multivariate statistical analysis was performed using the metaX software package, including principal component analysis (PCA) and supervised orthogonal partial least squares-discriminant analysis (OPLS-DA). The screening criteria for differential metabolites were: VIP≥1.0, Fold Change≥1.2 or ≤0.83, and *P* < 0.05 (where *P* represents the statistical significance determined by Student’s t-test for normally distributed data or Mann-Whitney U test for non-normally distributed data). Metabolic pathway enrichment analysis was conducted through HMDB and KEGG databases.

Correlation analysis between key differential metabolites and clinical indicators was performed using GraphPad Prism 10.1.2 software, calculating Spearman correlation coefficients (*r*) to evaluate the degree of association between disease severity, skin physiological parameters, and key differential metabolites.

## 3 Results

### 3.1 Clinical characteristics of study subjects

In this study, we conducted statistical analysis of the clinical characteristics of 17 ETR cases, 17 PPR cases, and 16 HC subjects included, as shown in Table 1. There were no statistically significant differences in gender, age, or BMI among the three groups (*P* > 0.05).

**TABLE 1:** Clinical characteristics of study subjects.

	ETR	PPR	HC	*P*
Number (person)	17	17	16	
Gender	15 female (2 male)	16 female (1 male)	12 female (4 male)	>0.05
Age (years)	34.18 ± 9.94	35.82 ± 7.87	35.06 ± 9.44	>0.05
BMI(kg/m2)	22.57 ± 2.51	22.78 ± 2.40	21.73 ± 2.31	>0.05
Disease duration (year)	4.0 (2.0–4.5)	3.0 (1.0–4.0)	-	>0.05
Skin hydration	66.11 ± 7.95	63.47 ± 9.22	69.50 ± 9.17	>0.05
TEWL	18.0 (13.0–24.0)	17.0 (14.0–20.0)	12.87 ± 5.12	<0.05
Sebum	85.0 (60.0–110.0)	86.0 (58.0–108.0)	49.5 (39.5–79.5)	>0.05
CEA				>0.05
1	2	3	-	
2	4	7	-	
3	5	4	-	
4	6	3	-	

Age, BMI, and skin hydration showed normal distribution and are expressed as mean ± standard deviation; disease duration, sebum, and TEWL, showed skewed distribution and are expressed as median (lower quartile ∼ upper quartile).

Regarding skin barrier function assessment, although skin hydration in the HC group was higher than in both rosacea subtypes, the difference was not significant (*P* > 0.05). TEWL showed an increasing trend in rosacea patients (*P* < 0.05), suggesting the presence of skin barrier disruption in rosacea patients. Notably, sebum secretion levels in the rosacea group were elevated compared to the healthy control group. Although the difference did not reach statistical significance (*P* > 0.05), this result still suggests that sebum metabolism abnormalities may be one of the key characteristics of this disease. Further analysis comparing the two rosacea subtypes revealed no significant differences between ETR and PPR in TEWL, skin hydration, or sebum content (*P* > 0.05), suggesting that barrier dysfunction in both subtypes shares common characteristics.

### 3.2 Metabolomics quality control

**FIGURE 1:**
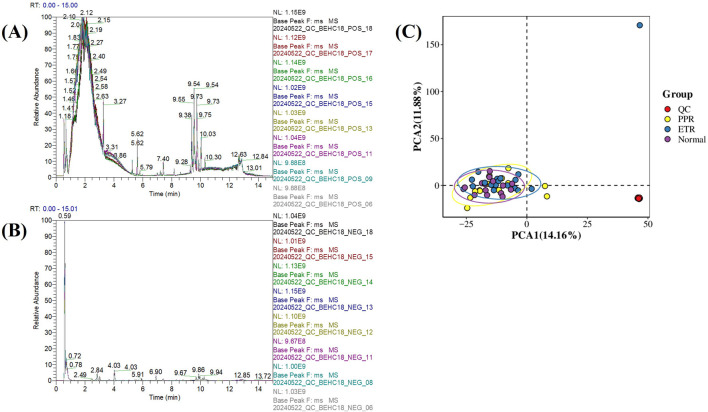
Metabolomics Quality Control Chart. Note: Figure **(A)** Base peak chromatogram of QC samples in positive ionization mode; Figure **(B)** Base peak chromatogram of QC samples in negative ionization mode (x-axis represents retention time, y-axis represents the response intensity of the strongest signal ion captured at each time point); Figure **(C)** Principal component analysis of all samples (x-axis represents the first principal component PCA1, y-axis represents the second principal component PCA2, with numbers in parentheses representing the scores of each principal component, indicating the percentage of overall variance explained by the corresponding principal component. Each point in the PCA score plot represents one sample, different groups are marked with different colors, and ellipses represent 95% confidence intervals).

The base peak chromatogram (BPC) of QC samples is a continuous plot obtained by depicting the intensity of the highest ion captured at each time point during QC sample detection. When all QC samples in positive and negative ionization modes were overlaid respectively, the high overlap of chromatographic peak response intensity and retention time indicated good instrument stability during the detection process and good data quality (see Figures 1A,B).

Principal component analysis (PCA) was performed on the quantitative values of all samples including QC in the overall dataset to observe the general distribution of each group of samples and QC samples. The results showed that QC samples clustered tightly, indicating small systematic error, good experimental reproducibility, and good data quality (see Figure 1C).

### 3.3 Stability analysis of metabolomics experimental results

**FIGURE 2:**
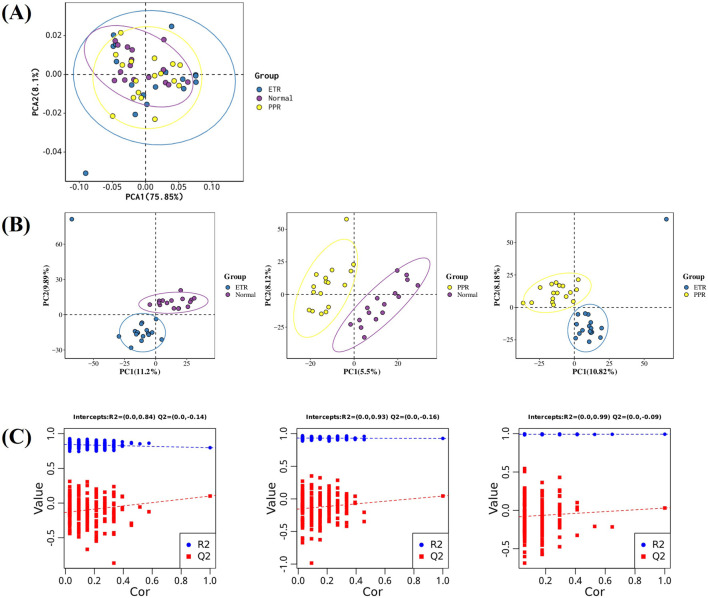
Multi-group Differential Analysis Plot. Note: Figure **(A)** PCA plot of ETR, PPR, and healthy controls (The x-axis represents the first principal component PC1, the y-axis represents the second principal component PC2, and the numbers in parentheses are the scores of each principal component, indicating the percentage of overall variance explained by the corresponding principal component.); Figure **(B)** OPLS-DA plot of ETR, PPR, and healthy controls (The x-axis Tscore represents the predictive principal component score of the first principal component, and the y-axis OrthogonalTscore represents the orthogonal principal component score.); Figure **(C)** 200-time response permutation test of the OPLS-DA model (*R* ^2^ represents the explained variance of the model, while Q^2^ represents the predictive ability of the model. The two points in the upper right corner represent the *R* ^2^ and Q^2^ values of the actual OPLS-DA model, while the points on the left represent the permutation test results obtained by randomly scrambling the Y-variable labels).

After LC-MS analysis and QC correction, PCA and OPLS-DA analyses were performed on blood samples from different rosacea subtypes and healthy controls. The results showed that the separation trend was not obvious under the unsupervised PCA model (see Figure 2A), suggesting that rosacea-related metabolic changes may be relatively complex and require more refined supervised analytical methods to further explore inter-group metabolic differences. The supervised OPLS-DA model showed separation trends, indicating that there are indeed characteristic metabolic pattern differences between different rosacea subtypes and healthy controls (see Figure 2B). To validate the reliability of the OPLS-DA model and exclude overfitting risks, we performed 200 response permutation tests (RPT) on the model (see Figure 2C). Our results showed that the Q^2^ intercept was negative, meeting basic requirements, but some random models overlapped with the original model, suggesting limited discriminant ability of the model. See Figure 2 for details.

### 3.4 Identification of differential metabolites between different rosacea subtypes and healthy control groups

**FIGURE 3:**
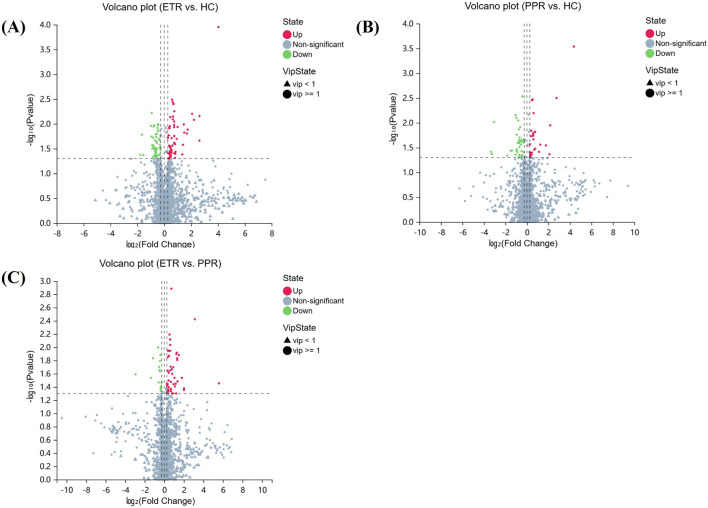
Volcano Plots of Differential Metabolites. Note: Figure **(A)** ETR group vs. HC group; Figure **(B)** PPR group vs. HC group; Figure **(C)** ETR group vs. PPR group. (The x-axis represents log2-transformed Fold Change, and the y-axis represents -log10-transformed p-value. Green indicates significantly downregulated differential metabolites, red indicates significantly upregulated differential metabolites, circles represent metabolites with VIP ≥1, triangles represent metabolites with VIP <1, and non-significant metabolites are shown in gray).

In combination with databases such as HMDB and KEGG, univariate and multivariate analyses were used to screen for differential metabolites between groups. Differential metabolites were identified through the following criteria: 1) VIP ≥1 in the OPLS-DA model, 2) Fold Change ≥1.2 or ≤0.83, and 3) *P* < 0.05. There were 114 metabolites with significant differences between ETR and healthy controls (63 upregulated, 51 downregulated); 64 metabolites showed significant differences between PPR and healthy controls (49 upregulated, 15 downregulated); and 64 metabolites exhibited significant differences between ETR and PPR (49 upregulated, 15 downregulated), as shown in Table 2. Volcano plots (Figure 3) intuitively display the differential metabolites between patients with different rosacea subtypes and healthy individuals.

**TABLE 2:** Statistical summary of differential metabolites.

Comparison group	Diff of total	Up	Down
ETR-vs-HC	114	63	51
PPR-vs-HC	64	28	36
ETR-vs-PPR	64	49	15

### 3.5 Metabolic pathway enrichment analysis of differential metabolites between ETR group and HC group

Differential metabolites between ETR and healthy controls were mainly concentrated in carbon metabolism, cholesterol metabolism, glycerolipid metabolism, antifolate resistance, aminoacyl-tRNA biosynthesis, alanine, aspartate and glutamate metabolism, vitamin B6 metabolism, pentose phosphate pathway, and central carbon metabolism in cancer. Notably, succinic semialdehyde (SSA) and 2,3-dihydroxypropanoic acid (2,3-DHPA) were significantly upregulated in carbon metabolism, while taurochenodeoxycholic acid (TCDCA) was significantly downregulated in cholesterol metabolism, and methionine (Met) was downregulated in multiple metabolic pathways. Results are shown in Table 3.

**TABLE 3:** Metabolic pathway enrichment analysis of differential metabolites between ETR group and HC group.

Pathway	Trend	Count2	Count all	P value_enrich	KEGG names	logP value
Carbon metabolism	Up	2	114	0.005863029	SSA; 2,3-DHPA	5.139088915
Cholesterol metabolism	Down	1	6	0.006086627	TCDCA	5.101661209
Glycerolipid metabolism	Up	1	14	0.0141479	2,3-DHPA	4.258189076
Antifolate resistance	Down	1	17	0.01715499	Met	4.065466186
Aminoacyl-tRNA biosynthesis	Down	1	24	0.02413797	Met	3.72396916
Alanine, aspartate and glutamate metabolism	Up	1	28	0.02810726	SSA	3.571727373
Vitamin B6 metabolism	Up	1	29	0.02909721	SSA	3.537112986
Mineral absorption	Down	1	29	0.02909	Met	3.537112986
Pentose phosphate pathway	Up	1	36	0.03600	2,3-DHPA	3.324228285
Central carbon metabolism in cancer	Down	1	37	0.03698	Met	3.297305854

This table shows the significantly enriched metabolic pathways of differential metabolites between ETR, and HC, groups, displaying the regulation direction, number of metabolites, and corresponding enrichment statistical data.

### 3.6 Metabolic pathway enrichment analysis of differential metabolites between PPR group and HC group

Differential metabolites in PPR patients were mainly associated with tryptophan metabolism, linoleic acid metabolism, serotonergic synapse, glycine, serine and threonine metabolism, and phenylalanine metabolism. In these pathways, 12-hydroxystearic acid (12-HSA) and 5-hydroxytryptophan (5-HTP) were enriched in tryptophan metabolism, with 12-HSA significantly upregulated and 5-HTP significantly downregulated in PPR patients. Dihomo-γ-linolenic acid (DGLA) was upregulated in linoleic acid metabolism. 5-Aminolevulinic acid (5-ALA) was upregulated in glycine, serine and threonine metabolism. 3-(3-Hydroxyphenyl)propanoic acid (3-HPPA) was downregulated in phenylalanine metabolism. Results are shown in Table 4.

**TABLE 4:** Metabolic pathway enrichment analysis of differential metabolites between PPR group and HC group.

Pathway	Trend	Count2	Count all	P value_enrich	KEGG names	logP value
Tryptophan metabolism	Down	2	83	0.001833219	12-HAS ;5-HTP	6.301681841
Linoleic acid metabolism	Up	1	27	0.02080048	DGLA	3.872779216
Serotonergic synapse	Down	1	41	0.03142798	5-HTP	3.4600567
Glycine, serine and threonine metabolism	Up	1	44	0.03369141	5-ALA	3.39051237
Phenylalanine metabolism	Down	1	49	0.03745293	3-HPPA	3.284670334

This table displays the metabolic pathway enrichment analysis of differential metabolites between PPR, and HC, groups, showing the regulation direction of metabolites, the number of differential metabolites in each metabolic pathway, and their enrichment statistical data.

### 3.7 Metabolic pathway enrichment analysis of differential metabolites between ETR group and PPR group

Key differential metabolic pathways between ETR and PPR groups include steroid hormone biosynthesis and ovarian steroidogenesis. Significant metabolites such as 5-androstenediol, ethanolamine glucuronide, and 16α-hydroxyestrone were enriched in the steroid hormone biosynthesis pathway, among which 5-androstenediol (5-AD) and etiocholanolone glucuronide (E.G.,) were upregulated, while 16-hydroxyestrone (16-OHE) was downregulated. 5-AD was also upregulated in ovarian steroidogenesis. Results are shown in Table 5.

**TABLE 5:** Metabolic pathway enrichment analysis of differential metabolites between ETR group and PPR group.

Pathway	Trend	Count2	Count all	P value_enrich	KEGG names	logP value
Steroid hormone biosynthesis	Up	3	99	0.0000321112	5-AD ;E.G., ;16-OHE	10.346305679
Ovarian steroidogenesis	Up	1	23	0.01503418	5-AD	4.197429003

This table displays the metabolic pathway enrichment analysis of differential metabolites between ETR, and PPR, groups, showing the regulation direction of metabolites, the number of differential metabolites in each metabolic pathway, and their enrichment statistical data.

### 3.8 Screening of key differential metabolites

For further analysis, metabolites meeting the criteria of FC ≥ 1.2 or ≤0.83, VIP ≥1, *P* < 0.05, and closely related to relevant metabolic pathways were identified as key differential metabolites among the comparison groups. Compared with the HC group, ETR group showed upregulation of SSA and 2,3-DHPA, and downregulation of TCDCA and Met. PPR group showed upregulation of 12-HSA, DGLA, and 5-ALA, and downregulation of 3-HPPA and 5-HTP. Compared with the PPR group, ETR group showed upregulation of 5-AD and, E.G., and downregulation of 16-OHE. Details are shown in Table 6.

**TABLE 6:** Key differential metabolites among ETR group, PPR group, and HC group.

Group	Differential metabolites	State	*P* value	VIP value	log2FC value
ETR vs. HC	Succinic semialdehyde	Up	0.012	1.035	0.353
ETR vs. HC	2,3-Dihydroxypropanoic acid	Up	0.048	1.719	0.435
ETR vs. HC	Taurochenodeoxycholic acid	Down	0.042	2.619	−1.547
ETR vs. HC	Methionine	Down	0.011	1.664	−0.460
PPR vs. HC	12-Hydroxystearic acid	Up	0.048	1.561	0.324
PPR vs. HC	Dihomo-γ-linolenic acid	Up	0.048	2.104	0.361
PPR vs. HC	5-Aminolevulinic acid	Up	0.003	1.841	0.448
PPR vs. HC	3-(3-Hydroxyphenyl)propanoic acid	Down	0.036	2.39	−1.38
PPR vs. HC	5-Hydroxytryptophan	Down	0.042	1.806	−0.452
ETR vs. PPR	5-Androstenediol	Up	0.009	2.432	0.602
ETR vs. PPR	Etiocholanolone glucuronide	Up	0.045	1.719	0.407
ETR vs. PPR	16-Hydroxyestrone	Down	0.025	2.945	−2.947

### 3.9 Confidence grading of key differential metabolites

To ensure the reliability of metabolite identification, we performed confidence level classification for all differential metabolites according to MSI guidelines. In this study, metabolite identification was primarily based on high-resolution mass spectrometry database matching, with identification results classified as Level 2 (probable metabolites, based on high-quality MS/MS spectrum matching with databases) and Level 3 (putative candidate metabolites, based on mass matching and limited MS/MS information). It should be noted that Level 2 and Level 3 identification results cannot distinguish stereoisomers and geometric isomers. Detailed identification information for all metabolites, including molecular formula, mass-to-charge ratio, retention time, MS/MS matching score, and confidence level, can be found in Table 7.

**TABLE 7:** Confidence Grading of key differential metabolite**s**.

Name	Formula	CalcMz	RT	Match	Level	Database
Succinic semialdehyde	C4H6O3	101.024	0.622	86.231	level2	mzCloud
2,3-Dihydroxypropanoic acid	C3H6O4	105.019	0.639	43.615	level3	mzCloud
Taurochenodeoxycholic acid	C26H45NO6S	498.288	8.263	98.904	level2	BMDB
Methionine	C5H11NO3S	150.058	1.146	38.775	level3	BMDB
12-Hydroxystearic acid	C18H36O3	299.259	9.262	89.609	level2	BMDB
5-Hydroxytryptophan	C11H12N2O3	221.092	2.846	10.058	level3	mzCloud
Dihomo-γ-linolenic acid	C20H34O2	305.248	10.117	99.918	level2	mzCloud
5-Aminolevulinic acid	C5H9NO3	132.067	0.811	3.423	level3	BMDB
3-(3-Hydroxyphenyl)propanoic acid	C9H10O3	165.056	2.812	93.094	level2	BMDB
5-Androstenediol	C19H30O5S	369.174	7.084	95.852	level2	BMDB
Etiocholanolone glucuronide	C25H38O8	465.2486	7.284	92.247	level2	BMDB
16-Hydroxyestrone	C18H22O3	287.164	8.598	9.869	level3	mzCloud

CalcMz: mass-to-charge ratio m/z; Formula: molecular formula; RT: retention time, unit min; Match: MS/MS, spectrum score; Database: database source for identification results; Level: confidence level of metabolite identification results.

### 3.10 Correlation analysis of key differential metabolites

**FIGURE 4:**
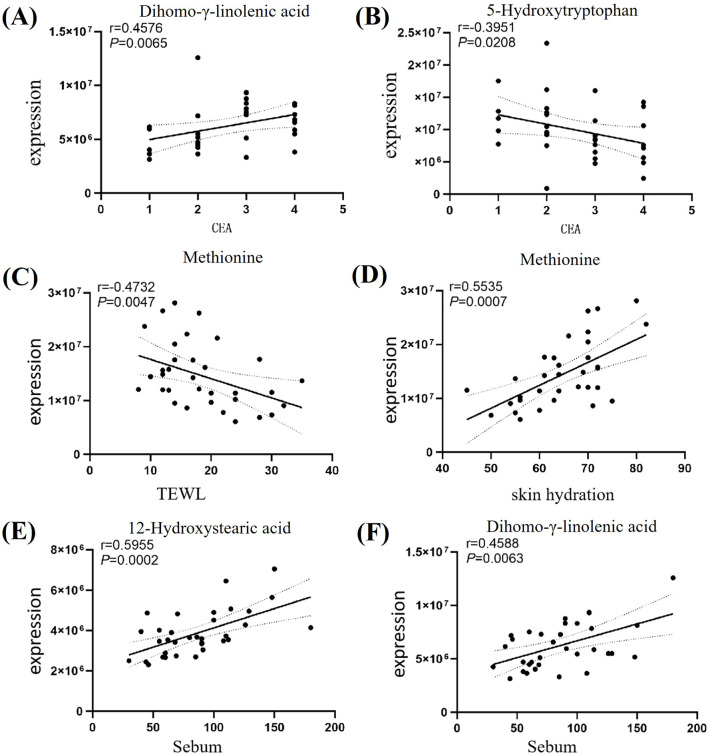
Differential Metabolites Association Network. Note: Figure **(A)** Correlation analysis between DGLA and CEA; Figure **(B)** Correlation analysis between 5-HTP and CEA; Figure **(C)** Correlation analysis between Met and transepidermal water loss; Figure **(D)** Correlation analysis between Met and skin hydration; Figure **(E)** Correlation analysis between 12-HSA and oil content; Figure **(F)** Correlation analysis between DGLA and oil content.

Spearman correlation analysis between the screened key differential metabolites and disease severity (CEA score) and skin physiological parameters (transepidermal water loss, skin hydration, oil content) revealed: DGLA was positively correlated with disease severity (*r* = 0.4576, *P* = 0.0065), while 5-HTP was inversely correlated with disease severity (*r* = −0.3951, *P* = 0.0208). Met was positively correlated with skin hydration (*r* = 0.5535, *P* < 0.0208) and negatively correlated with transepidermal water loss (*r* = −0.4732, *P* < 0.0047). Both 12-HSA (*r* = 0.5955, *P* = 0.0002) and DGLA (*r* = 0.4588, *P* = 0.0063) were positively correlated with skin oil content. Details are shown in Figure 4.

### 3.11 ROC analysis of key differential metabolites

**FIGURE 5:**
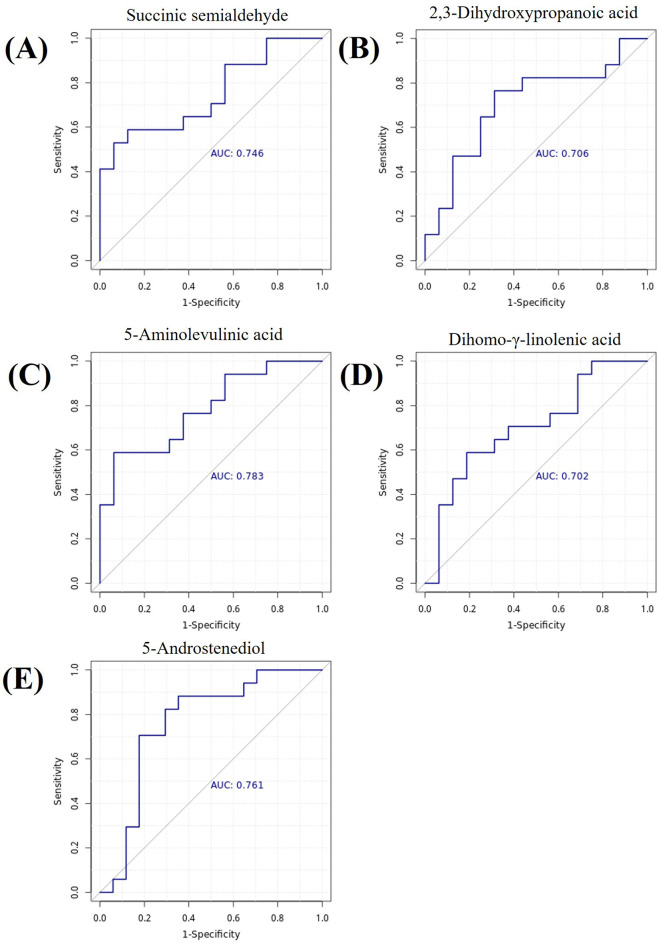
Metabolite ROC Curves. **(A)** Succinic semialdehyde, AUC = 0.746. **(B)** 2,3-Dihydroxypropanoic acid, AUC = 0.706. **(C)** 5-Aminolevulinic acid, AUC = 0.783. **(D)** Dihomo-γ-linolenic acid, AUC = 0.702. **(E)** 5-Androstenediol, AUC = 0.761. Note: The x-axis represents 1-specificity, and the y-axis represents sensitivity. The area under the curve is the AUC value.

To evaluate the discriminative ability of key differential metabolites for rosacea subtypes, we further performed receiver operating characteristic (ROC) curve analysis. The area under the ROC curve (AUC) is an indicator for measuring the diagnostic efficacy of metabolites. The higher the AUC value, the stronger the discriminative ability of the metabolite as a potential biomarker. The analysis results showed that multiple metabolites exhibited good diagnostic potential (AUC >0.7). See Figure 5.

## 4 Discussion

Rosacea is a chronic inflammatory skin disease that primarily affects the central facial region, with symptoms including erythema, papules, pustules, and telangiectasia. Its pathogenesis is complex, involving interactions between genetic susceptibility, immune dysregulation, neurovascular instability, and environmental factors (van Zuuren et al., 2021). In recent years, advances in metabolomics have provided unprecedented insights into metabolic disorders associated with rosacea, thereby helping to identify potential biomarkers and therapeutic targets (Liu et al., 2022). This study, through integrating clinical feature analysis with untargeted metabolomics data, revealed the pathophysiological characteristics of rosacea and its potential metabolic regulatory mechanisms.

### 4.1 Metabolites in ETR patients and their clinical significance

#### 4.1.1 Neurotransmitter metabolism and neuropsychiatric symptoms

In ETR patients, an important finding was the elevation of succinic semialdehyde (SSA) levels, which is an intermediate product in the gamma-aminobutyric acid (GABA) metabolic pathway. SSA accumulation inhibits GABA transaminase activity, leading to elevated GABA levels (Grover et al., 2024). GABA is an inhibitory neurotransmitter, and its serum elevation is closely associated with depressive symptoms (Bhandage et al., 2019). This finding is consistent with clinical observations of depression comorbidity in rosacea patients (Chang et al., 2022), suggesting that SSA disruption may be a potential cause of neurological symptoms in rosacea.

#### 4.1.2 Vascular regulatory substances and skin erythema symptoms

2,3-dihydroxypropanoic acid (2,3-DHPA) was similarly elevated in ETR patients. Previous studies have found that 2,3-DHPA can act on vascular smooth muscle and cause vasodilation (Luo et al., 2024). This finding provides an important metabolic basis for the characteristic paroxysmal flushing and persistent erythema in ETR patients, suggesting that elevated blood 2,3-DHPA levels may be a direct trigger for their vascular symptoms.

#### 4.1.3 Anti-inflammatory bile acids and inflammatory regulation imbalance

Taurochenodeoxycholic acid (TCDCA), a bile acid with anti-inflammatory properties, was significantly decreased in ETR patients. TCDCA primarily regulates inflammatory responses by activating G protein-coupled bile acid receptor 5 and glucocorticoid receptors (Bao et al., 2021), inhibiting pro-inflammatory cytokines such as TNF-α and IL-1β while enhancing the expression of anti-inflammatory cytokines such as IL-10 (Qi et al., 2020; Liu et al., 2011). The reduction of TCDCA may weaken the body’s anti-inflammatory capacity, thereby exacerbating the inflammatory response in rosacea.

#### 4.1.4 Amino acid metabolism and skin barrier function

Methionine (Met), an important sulfur-containing essential amino acid, also showed a declining trend in ETR patients. Correlation analysis revealed that Met was positively correlated with epidermal water content and negatively correlated with transepidermal water loss, indicating its close relationship with skin barrier function. After Met is converted to cysteine, it participates in disulfide bond formation in the skin epidermis (Rehman et al., 2020; Solano, 2020), and these disulfide bonds play a crucial role in maintaining keratin stability (Feng and Coulombe, 2015). Met is a precursor of glutathione (Wu et al., 2004), which serves as a key intracellular antioxidant molecule involved in free radical scavenging and detoxification processes (Liu et al., 2012; Morin et al., 2019). Therefore, Met deficiency may impair skin barrier function through dual mechanisms: on one hand, directly affecting the structural stability of stratum corneum proteins, and on the other hand, weakening the skin’s defense capacity against oxidative stress, making the skin more susceptible to external stimuli that trigger inflammatory responses.

### 4.2 Metabolites in PPR patients and their clinical significance

#### 4.2.1 Anti-inflammatory fatty acids and compensatory repair mechanisms

12-hydroxystearic acid (12-HSA) showed an elevated trend in the blood of PPR patients. 12-HSA is a commonly used bioactive substance in skincare that can inhibit interleukin-1α (IL-1α) release and exert anti-inflammatory effects (Mi et al., 2019). Its elevation may reflect the operation of the body’s negative feedback regulatory mechanism, representing an attempt at anti-inflammatory self-repair. Additionally, this study found that 12-HSA was positively correlated with skin surface oil content in rosacea patients, suggesting that this hydroxylated long-chain fatty acid (Dari et al., 2023) may play an important role in skin surface lipid metabolism in PPR patients.

#### 4.2.2 Photosensitive metabolites and skin symptoms

5-aminolevulinic acid (5-ALA) also showed significant elevation in PPR patients. 5-ALA is a photosensitizer precursor that can be converted to highly photosensitive protoporphyrin IX under specific wavelength light activation (Kelty et al., 2002). Previous studies have shown that facial skin of rosacea patients exhibits certain photosensitivity (McCoy, 2020; Zhou and Yin, 2024). Elevated blood 5-ALA levels may further amplify this photodamage effect, leading to skin burning sensation, worsening erythema, and even triggering acute episodes, providing a metabolic explanation for photosensitive symptoms.

#### 4.2.3 Polyunsaturated fatty acids and vascular and inflammatory responses

Dihomo-γ-linolenic acid (DGLA) is an ω-6 series polyunsaturated fatty acid (PUFA) that also showed an upregulated trend in PPR patients. DGLA has dual biological effects: on one hand, it exerts anti-inflammatory and vasodilatory effects by metabolizing through the cyclooxygenase pathway to 1-series prostaglandins (particularly PGE1) or by converting through the 5-lipoxygenase pathway to 15-(S)-hydroxy-8,11,13-eicosatrienoic acid (Sergeant et al., 2016). On the other hand, DGLA can be further converted by Δ5-desaturase to arachidonic acid (ARA) to exert pro-inflammatory effects (M et al., 2023). Due to limited Δ5-desaturase activity, normally only a small portion of DGLA is converted to ARA (M et al., 2023). Rosacea patients may have activated Δ5-desaturase activity, promoting DGLA conversion to ARA. ARA can generate prostaglandin E2 through the cyclooxygenase pathway to promote erythema and telangiectasia, and can also generate leukotriene B4 through the 5-lipoxygenase pathway to drive neutrophil infiltration, forming the characteristic pustules in PPR. Previous studies have found that DGLA is related to the body’s inflammatory state, with plasma DGLA levels positively correlated with high-sensitivity C-reactive protein, chemokine 5, interferon-γ, interferon-γ-induced protein 10, and platelet-derived growth factor b (Perreault et al., 2014), further reflecting the inflammatory characteristics of PPR. Elevated blood DGLA levels are associated with obesity, type 2 diabetes, liver dysfunction, Crohn’s disease, celiac disease, and other conditions (Mustonen et al., 2023), further highlighting the systemic characteristics of rosacea.

#### 4.2.4 Neurotransmitter precursors and emotional regulation

5-hydroxytryptophan (5-HTP) is a direct precursor of the neurotransmitter serotonin (Turner et al., 2006). 5-HTP levels were decreased in PPR patients. Serotonin participates in regulating various physiological processes including mood and sleep (Švob Štrac et al., 2016; Singh et al., 2023). Decreased 5-HTP levels may lead to impaired serotonin synthesis, explaining the common emotional disorders and sleep disturbances in rosacea patients. Beyond neuropsychiatric effects, 5-HTP also has multiple biological functions. Rondanelli et al. found that appropriate 5-HTP supplementation could increase satiety and reduce BMI in obese patients (Rondanelli et al., 2012). Additionally, 5-HTP has potential for treating inflammatory diseases and oxidative stress. Studies have found that 5-HTP also exhibits free radical scavenging activity (Keithahn and Lerchl, 2005) and has significant protective effects against hyperglycemia-induced oxidative stress (Derlacz et al., 2007). The Chae team discovered that 5-HTP can inhibit the release of inflammatory mediators nitric oxide (NO) and IL-6 (Chae et al., 2009). Therefore, decreased 5-HTP levels may simultaneously affect the nervous system, metabolic regulation, and inflammatory control (Maffei, 2020), providing a combined explanation for the systemic symptoms of rosacea and suggesting that exogenous 5-HTP supplementation may become a potential strategy for alleviating rosacea symptoms.

#### 4.2.5 Gut microbiome metabolites and the gut-skin axis

3-(3-hydroxyphenyl)propanoic acid (3-HPPA) also showed a declining trend in PPR patients. 3-HPPA is an important metabolite produced by gut microbiota through the metabolism of dietary polyphenolic compounds (Ono et al., 2020). Numerous studies have confirmed that the gut microbiome is closely related to skin health (Mahmud et al., 2022). The reduction of 3-HPPA as a microbial metabolite may reflect gut microbiota dysbiosis, which subsequently affects skin health. Future research is needed to clarify the role of 3-HPPA in the pathogenesis of rosacea.

This study revealed subtype-specific metabolite alterations in different rosacea subtypes through metabolomics analysis. These findings explain the clinical manifestations of rosacea from multiple perspectives: neurotransmitter metabolism disorders (elevated SSA, decreased 5-HTP) may be associated with neuropsychiatric symptoms; abnormal vasoactive substances (elevated 2,3-DHPA, altered DGLA metabolism) may lead to characteristic vascular symptoms; decreased multiple anti-inflammatory substances (TCDCA, Met, 5-HTP) and elevated photosensitive substances (5-ALA) may exacerbate inflammatory responses; changes in gut microbial metabolites (3-HPPA) suggest the potential role of the gut-skin axis in disease development. These metabolite alterations not only explain the local skin symptoms of rosacea but also provide a metabolic basis for its systemic characteristics and common comorbidities. Future research should further validate the diagnostic value of these metabolites as biomarkers and explore targeted intervention strategies based on metabolic pathways, providing new directions for precision diagnosis and treatment of rosacea.

### 4.3 Limitations

This study preliminarily revealed the complex interactions among amino acid, lipid, and hormone metabolism in rosacea patients, but several limitations remain. First, the limited sample size restricts the generalizability and statistical power of the results to some extent, requiring validation in larger independent cohorts in the future. Second, the high proportion of females in this study population, combined with the lack of recording of menstrual cycle phases and hormonal contraceptive use among female volunteers, may introduce confounding bias, particularly affecting the interpretation of steroid hormone-related metabolite levels. Therefore, future studies need to further optimize cohort design, balance gender ratios, and systematically record hormone-related status. Additionally, the associations between metabolites and pathological mechanisms proposed in this study are primarily based on bioinformatics analysis and literature evidence, and have not yet been validated through targeted metabolomics techniques or *in vitro*/*in vivo* functional experiments. Future research needs to combine multi-omics technologies with experimental models to thoroughly investigate the specific functional roles of these metabolic changes in the occurrence and development of rosacea.

## 5 Conclusion

This study revealed complex interactions between amino acid, lipid, and hormone metabolism in rosacea. These findings elucidated the metabolic characteristics of rosacea and their associations with disease severity and skin physiological parameters, providing new theoretical foundation and potential targets for subtype diagnosis and precision treatment of the disease. However, the small sample size limited the generalizability of the research results, and future validation in larger-scale cohorts is needed.

## Data Availability

The original contributions presented in the study are included in the article/Supplementary Material, further inquiries can be directed to the corresponding author.
